# Strategies for Conducting Blended Learning in VET: A Comparison of Award-Winning Courses and Daily Courses

**DOI:** 10.3390/bs15060787

**Published:** 2025-06-06

**Authors:** Yiran Cui, Meng Li, Yangyang Luo

**Affiliations:** 1 College of International Education, Shandong University, Jinan 250100, China; cuiyiran@sdu.edu.cn; 2 Institute for Advanced Studies in Education, Shandong University, Jinan 250100, China; 3 China National Academy of Educational Sciences, Beijing 100088, China; 4 Institute of Education, Tsinghua University, Beijing 100084, China; 5 Institute of Higher Education, Lanzhou University, Lanzhou 730000, China

**Keywords:** blended learning, instructional strategies, vocational education, video analysis

## Abstract

Blended learning has emerged as a popular trend in education; however, as students from higher vocational colleges who are often in the lower 50% of national standardized entrance exam achievers, their teachers face unique challenges in implementing blended learning. This study summarized effective blended learning strategies in higher vocational education and training (VET) classrooms by comparing 53 award-winning and 40 daily course videos in China. Firstly, video analysis and Lag sequential analysis were employed to identify effective strategies for implementing blended learning in diverse VET course types. A set of general and specific blended learning strategies was developed to help VET teachers adapt instructional approaches accordingly. Secondly, a questionnaire survey among 215 VET teachers revealed positive perceptions of the strategies in terms of usability, ease of use, perceived behavioral control, and intention to use them. The present research provides valuable guidance for VET teachers to effectively implement blended learning strategies in diverse course types, contributing to the understanding of effective blended learning strategies in VET and addressing the gap in research for this unique teaching stage.

## 1. Introduction

With the deep integration of various digital technologies into education, especially following the impact of COVID-19, blended learning, which combines the strengths of online and offline instruction (Garrison & Kanuka, 2004), is rapidly spreading and becoming normalized globally (Bruggeman et al., 2021; Ma et al., 2025). While extensive research exists on blended learning, its application within China’s vocational education and training (VET) classrooms remains underexplored.

Previous studies indicate that VET teachers perceive blended learning as a potential solution to challenges such as limited classroom time, heterogeneous student academic levels, and over-reliance on teacher guidance (Bliuc et al., 2012; H. Chen & He, 2020; Xu, 2021). However, vocational education in China is also characterized by unique learners. VET students are in the latter half of preparing for China’s college entrance examination (Stewart, 2015), so they are often considered to lack academic ability (Hao & Pilz, 2021; Wang, 2021). Consequently, the self-regulated learning emphasized in blended learning may be challenging to implement. VET teachers may often mistakenly believe they are successfully implementing blended learning when they are not. An analysis of the texts submitted by 122 Chinese VET teachers revealed that many teachers lack a clear understanding of blended learning, viewing it merely as a combination of online and offline activities or in-class and extracurricular activities; naturally, it is challenging to achieve the desired effect of blended learning (Cui et al., 2022). Moreover, although VET courses differ in terms of content and objectives, few studies have examined appropriate blended instructional strategies for different courses. Therefore, exploring effective strategies to guide VET teachers in blended learning is essential.

Award-winning courses offer possible insight into these challenges (Kumar et al., 2019; Song, 2022). In recent years, VET teaching competitions in China have mandated blended learning elements, requiring that teaching videos reflect actual and complete content (Ministry of Education of the People’s Republic of China, 2020). Consequently, national award-winning courses represent the experts’ appreciation for curriculum patterns and provide a reference for VET teachers on implementing blended learning effectively. By comparing the teaching and learning behaviors observed in award-winning and daily course videos, this study aims to identify key differences and generate effective strategies to guide VET teachers in implementing blended learning within routine teaching contexts.

## 2. Literature Review

### 2.1. Strategies for Conducting Blended Learning in VET

Instructional strategies are specific methods to guide activities toward particular goals and instructional contexts, and events composed of behavioral sequences are the core structure of instructional strategies (Gibbons, 2020). Blended learning strategies are often proposed through frameworks such as the community of inquiry (COI, X. Feng et al., 2017; Ma et al., 2025), self-determination theory (SDT, Deci & Ryan, 1987; Ameloot et al., 2024), and self-regulated learning theory (Eggers et al., 2021; Luo & Zhou, 2024).

Strategies recommended by previous studies include granting students autonomy in task selection (Anthonysamy et al., 2020), establishing a clear link between classroom and extracurricular activities (Thai et al., 2017), creating a conducive atmosphere (Ma et al., 2015; Vaughan, 2015), organizing tasks based on learners’ existing cognitive level, and providing sufficient time to complete tasks (Kim et al., 2014). Additionally, scholars have also emphasized encouraging anonymous discussions among students (Miyazoe & Anderson, 2011), offering clear guiding scaffolds (Bernard et al., 2014), providing timely incentives (Jayalath & Esichaikul, 2020), and fostering self-reflection among learners and peer evaluation (Luo & Zhou, 2024). The emotional interaction and communication among teachers, students, and peers also are emphasized (Ma et al., 2015). However, while these approaches are well-documented in higher education, their adaptation to VET remains underexplored. Few studies have focused on the strategies suitable for VET teachers to implement blended learning.

### 2.2. Video Analysis in Evidence-Based Education Research

Evidence-based education has recently gained significant attention among researchers and practitioners (Gorard et al., 2020; Ostinelli & Crescentini, 2021). Classroom video, which records the behavior of teachers and students, provide an intuitive representation of the teaching and learning process. Researchers can replay these videos to capture specific classroom interactions in greater detail, enhancing their understanding of the teaching dynamics (Barron et al., 2013). TALIS (Teaching and Learning International Survey) and TIMSS (Trends in International Mathematics and Science Study) include classroom videos from various countries, vividly illustrating classroom practice and promoting in-depth discussions and reflections among researchers and practitioners (Jerrim, 2024; Sikorski & Straus, 2025). In recent years, many studies have quantified typical classroom behaviors using Lag Sequential Analysis (LSA), a method often used to analyze complex interactions by discovering sequences of behaviors that occur more frequently than by chance (Sackett, 1978; Tan et al., 2022). An increasing number of studies have recently employed LSA to analyze the behavioral sequences of both teachers and students in classroom videos, revealing the dialogue structures between them (S. Y. Wu, 2021; Song et al., 2023). For example, Cheng and Tsai (2019) used LSA to identify different behavioral patterns of teacher–student interactions during virtual field trips in an elementary classroom. Kucuk and Sisman (2017) used LSA to understand the behavioral patterns of elementary students and teachers in the one-to-one robotics instructional process and discussed these patterns in terms of gender differences and difficulty level of robotics activities. Song et al. (2023) conducted LSA to compare the dialogic patterns of mathematics lessons captured in elementary, middle, and high schools in China, using video-recorded lessons. In summary, revealing the process of teacher–student interaction through LSA enables evidence-based summarization of teaching strategies.

### 2.3. Research Gap and Present Study

Based on the above literature, blended learning has gained widespread acceptance among researchers and practitioners. However, several research gaps still require further exploration. First, in terms of participants, previous studies focus on undergraduate and graduate students in higher education, while less attention has been given to Chinese VET students, who are more skill-oriented and have lower academic levels. Second, most of the previous blended learning strategies have been proposed by researchers based on theoretical deductions and subjective experiences, ignoring the practical experiences of the actual users, i.e., teachers, especially experienced and commended award-winning teachers. Third, the strategies proposed in previous studies are often universal. There are notable differences among various VET courses, but few studies have attempted to classify these differences appropriately.

This study addresses these gaps by synthesizing video evidence and teacher feedback. The following research questions guide this study: What general blended learning strategies applicable to VET courses can be identified through a comparative analysis of blended learning videos in award-winning and daily courses? Which specific strategies are used for different types of VET courses? How effective are these strategies perceived by frontline VET teachers?

## 3. Methods

### 3.1. Design Process

**Figure 1:**
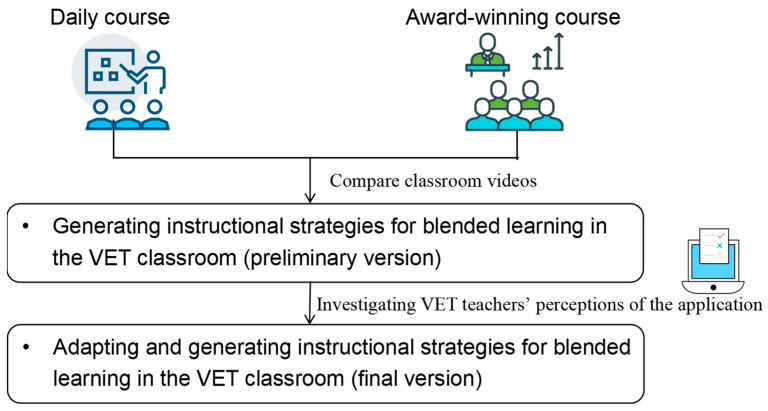
Research process in this study.

The present study analyzed teaching and learning behaviors from blended learning videos to identify and summarize effective instructional strategies. As shown in Figure 1, the current study first compared blended learning videos from 53 award-winning courses with those from 40 daily courses to analyze the differences in teachers’ and students’ behaviors presented in similar VET courses. Then, a questionnaire survey was administered to 215 VET teachers to assess how well these strategies are adopted and perceived as practical.

### 3.2. Datasets and Analysis of Classroom Videos

#### 3.2.1. Datasets

VET content can be divided into technical theoretical knowledge, which emphasizes cognitive development through teacher–student dialogue, and technical practical knowledge, which focuses on skill acquisition via teacher demonstrations and student-led operations (Brockmann et al., 2008; Orozco et al., 2019). Based on the above information, Cui et al. (2025) categorized Chinese VET courses into the three most common types: teacher–student dialogue (Type1), teacher demonstration (Type2), and student-independent operation (Type3). This study also adopted this classification and focused on these three types of courses.

The award-winning courses (n = 53) were sourced from the National Vocational Institutions Teaching Competition platform (retrieved from http://www.nvic.edu.cn/CompetitionVideo?type=5, accessed on 7 December 2022), a nationally organized competition in which the videos of the winning courses were recorded and available for free public access for non-commercial use. The daily courses (n = 40) were recorded during regular course sessions from four vocational colleges in China, and the scope of the recordings includes teacher and student behaviors. We contacted VET teachers and institution administrators, asking them to share volunteer recruitment information with their colleagues. The participants were informed of the purpose of the study before the video recording and were told that sending the video would be considered informed consent. The duration of the videos for the three types of award-winning and daily courses is shown in Table 1.

#### 3.2.2. Data Coding

The teacher and student behaviors in the video need to be coded so that the unstructured video can be transformed into a series of meaningful and informative coded labels (Tripp & Rich, 2012; G. Chen et al., 2020). Traditional coding system like the Flanders Interaction Analysis System (FIAS) uses three-second intervals as coding units, focusing on the verbal interactions between teachers and students (Flanders, 1963; Cai et al., 2023). However, since VET emphasizes hands-on skills, necessitating broader frameworks that incorporate non-verbal behaviors, such as operational demonstrations and task execution, a new coding framework was developed for the VET courses.

Interaction theory is the most commonly used theoretical basis for analyzing classroom interactions; thus, teaching and learning behaviors can be categorized into three types: teacher–student interaction, student–student interaction, and student–content interaction (Moore, 1989; Boon, 2021). Furthermore, according to the direction of the information flow between the interacting subjects, interactions can be divided into teacher-to-student, student-to-teacher, and teacher–student bidirectional interactions (Pica, 1987; Zografos, 2023). Additionally, the participants have one-to-one individual interaction and one-to-many social interaction (Jung et al., 2002; Jensen & Helles, 2017). The final coding frameworks for blended learning in the VET course are presented in Table 2.

After the coding framework was established, two researchers coded videos from the 24 award-winning lessons and the 20 daily lessons separately. The Cohen’s Kappa value of 0.86 (*p* < 0.001) confirmed that inter-rater reliability was satisfactorily met. Then, one of the researchers coded all subsequent lesson videos.

#### 3.2.3. Data Analysis of Classroom Videos

The Lag Sequential Analysis (LSA) method was used to examine the likelihood of one behavior occurring followed by another and its statistical significance (Sackett, 1978; Tan et al., 2022). For each type of course, the video coding of both award-winning courses and daily courses were analyzed using the LSA method. The Z-score, which represented the adjusted residual value, indicated the significance of sequences of behaviors. When the Z-score>1.96 (*p* < 0.05), it signified that the corresponding behavior sequences were statistically significant (Tlili et al., 2023). The Chi-square test was also employed to examine the differences in representative teaching behaviors across different courses, providing evidence for developing instructional strategies. GSEQ 5.1 and IBM SPSS Statistics for Windows, version 21.0 (IBM Corp, 2012), were used for data analysis.

### 3.3. Participants and Data Analysis of the Questionnaire

#### 3.3.1. Participants

The questionnaire survey targeted VET teachers of professional courses. Based on the principles of purposive and convenience sampling, participants were deliberately selected from nine vocational colleges located in four provincial administrative regions (i.e., Fujian, Guangdong, Shandong, and Xinjiang), which represent diverse geographical regions and varying levels of economic development. Administrators from each college selected 20–30 teachers who were familiar with blended learning to participate in the survey. The researcher distributed and collected the anonymous paper questionnaires in person to address any questions or suggestions from the participants in real time. The purpose of the survey was explained, and ethics commitments, such as voluntary participation and confidentiality, were ensured.

Initially, 215 questionnaires were collected. After excluding those with more than 20% missing data, a final valid sample size of 183 was obtained. The samples covered 14 out of the 19 major professional categories defined in China’s vocational education system. Depending on the type of course taught by the teacher, the numbers of the three types were 79, 61, and 43, respectively. Detailed information is presented in Table 3.

#### 3.3.2. Measurement

This survey explored the perceptions of Vocational Education and Training (VET) teachers regarding the proposed blended learning strategies using the Technology Acceptance Model (TAM), which is frequently employed to assess teachers’ acceptance and willingness to integrate information technology into their courses (Bin et al., 2020; Mailizar et al., 2021). Within the various frameworks of TAM, the classic Unified Theory of Acceptance and Use of Technology (UTAUT) model suggested that individuals’ cognitive beliefs about technology positively influence their usage behavior, with behavioral intentions acting as mediators (Venkatesh et al., 2003; Smirani & Boulahia, 2022). The subsequent models derived from TAM highlighted that perceived ease of use and perceived usefulness were central to the cognitive belief dimension (Yang et al., 2023). Furthermore, the Theory of Reasoned Action (TRA) and the Theory of Planned Behavior (TPB) indicated that perceived behavioral control can also be considered a cognitive belief (Ajzen & Fishbein, 1980; Ajzen, 1991; C. Su & Chao, 2022). In summary, five key research constructs were identified: perceived ease of use, perceived usefulness, perceived behavioral control, behavioral intention, and current usage behavior.

Considering that each strategy is illustrated in relation to a specific teaching scenario, it is convenient for teachers to directly understand and judge the perceived level of usability and ease of use. Participants rated the following statements for each strategy on a scale from 1 to 5, with 1 being “strongly disagree” and 5 being “strongly agree”: (1) I find the strategy easy to understand; (2) I find the strategy useful; (3) I believe I can use the strategy competently; (4) I would like to use the strategy; and (5) I have frequently used the strategy in blended learning. The questionnaire contained 80 questions in total, and Cronbach’s alpha coefficient was 0.966, which indicates good reliability. Additionally, the questionnaire included an optional open-ended item through which participants could provide feedback or suggestions on any of the strategies.

#### 3.3.3. Data Analysis of the Questionnaire

SPSS 21.0 was used to present the overall perceptions of VET teachers regarding each blended learning strategy proposed in this study.

## 4. Results

### 4.1. Results of General Blended Learning Strategies Constructed from Video Analysis

#### 4.1.1. General Strategies Based on Descriptive Statistical Results About Questioning

**Figure 2:**
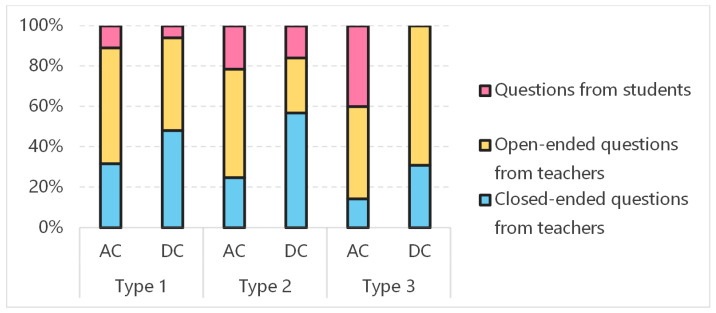
Time distribution of question-and-answer activities in various instructional videos. Note: AC is award-winning courses; DC is daily courses. Type1: teacher–student dialogue; Type2: teacher demonstration; Type3: student-independent operation.

Questioning is an important activity in teacher–student interaction, which can be classified according to the initiator (teacher/student) and cognitive demand (closed/open-ended) (Worley, 2015; Khoza & Msimanga, 2022). Closed-ended questions require specific answers, enabling direct assessment of students’ memory and often resulting in binary responses such as “yes” or “no”. They rarely encourage extended dialogue or follow-up prompts from the teacher. In contrast, open-ended questions do not have fixed answers and often prompt further discussion and critical thinking. Table 4 and Figure 2 illustrate the time distribution of three question-and-answer activities in various instructional videos from both award-winning and daily courses.

The results indicated that, regardless of the course type, the amount of time dedicated to “student questioning” was significantly higher in the award-winning videos than in the daily videos. Thus, it suggested that teachers in the award-winning courses prioritized providing students with opportunities to express and ask questions. Additionally, the questions they posed were predominantly open-ended, while closed-ended questions were more common in the daily videos. This trend indicated that teachers in the award-winning courses focused more on stimulating students’ thinking through open-ended questioning.

#### 4.1.2. General Strategies Based on LSA Results About Feedback

LSA enabled the construction of behavioral transition diagrams using significant Z-score thresholds (Z > 1.96) to visualize classroom interaction structures. These diagrams employ directed arrows to denote behavioral flow, with line thickness proportional to transition significance. Schematic representations for the three course categories (award-winning vs. daily courses) are provided in Appendix A.

As shown in Table 5, the Z-score results for the behavioral sequence TS2 to TS3 were significant for various award-winning videos but not statistically significant in the daily videos, indicating that teachers in award-winning courses prioritized presenting course content in the context of real-world application. In addition, TSG5 and TSG6 represented teachers providing cognitive and affective feedback on students’ performance, respectively. The Z-score results of TSG6 to TSG5 were significant in Type1 and 2, and the Z-score of TSG5 to TSG6 was significant in Type3. The interactions between these two types of feedback were significant in the award-winning videos but insignificant in the daily videos, indicating that teachers in the award-winning courses more frequently used encouraging language when providing feedback and tended to integrate cognitive guidance with emotional encouragement.

#### 4.1.3. Results on the General Blended Learning Strategies After Integration

Based on the analytical approach described above, the teaching and learning behaviors observed in the different types of courses presented in Appendix A were systematically compared to summarize instructional strategies. The results are shown in Table 6. The strategies were categorized into different teaching phases, eight of which apply to technology-supported classroom scenarios, and seven apply to face-to-face instruction. Additionally, we found that this series of instructional strategies can be explained using self-determination theory (SDT). Table 6 also presents the self-determination theory dimensions corresponding to each strategy, with further explanations provided in the discussion section.

### 4.2. Results of Specific Blended Learning Strategies Constructed from Video Analysis

#### 4.2.1. Specific Strategies Based on LSA Results About Knowledge Transfer

In addition to common instructional strategies, each of the three types of VET courses had specific strategies. For example, effective transmission of theoretical knowledge to students is essential in teacher–student dialogue courses (Yan, 2019; L. Feng et al., 2021). Key behaviors related to knowledge transfer include teacher states content (TS4), dialogue initiated by the teacher asking closed questions (TSG1), and dialogue initiated by the teacher asking open questions (TSG2). These three behaviors accounted for the majority of instructional time spent in the teacher–student dialogue-based classroom. Their percentages of time in the award-winning video were 15.8%, 11.3%, and 20.5%, while in the daily video, they were 25.5%, 13.8%, and 13.2%, respectively.

**Figure 3:**
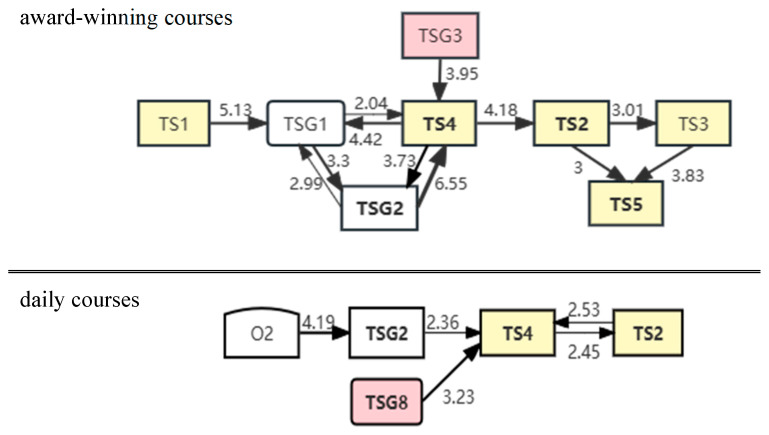
Behavioral sequences of teacher transfer knowledge part of the video in Type1. Note: Teacher operation (TS1); Teacher relates content to work context (TS2); Teacher expresses ideas about content (TS3); Teacher states content (TS4); Teacher states rules and action requirements (TS5); Authoritative discussion (teacher asks and answers themselves and students respond with yes or no) (TSG1); Teacher-led dialogue (teacher asks and students respond) (TSG2); Student-led dialogue (student ask and teacher responds) (TSG3); Teacher provides instructions for students to during operations (TSG8); Silence that does not contribute to the lesson (O2).

Figure 3 shows a graph of significant behavioral transitions related to the knowledge transfer component in a teacher–student dialogue-based course. As shown in Figure 3, the relationships among TS4, TSG1, and TSG2 in the award-winning videos were interrelated. In contrast, the daily videos showed that only TSG2 significantly pointed to TS4, reflecting the unidirectional approach adopted by teachers over a long period. Therefore, it was recommended that during the knowledge transfer phase of Type1, teachers should combine traditional information transmission with guided question-and-answer sessions. This approach would encourage students to participate in cognitive processing rather than passively receiving information.

#### 4.2.2. Results on the Specific Blended Learning Strategies After Integration

Action-oriented learning is emphasized in higher vocational education (Bünning, 2007; Hoekstra et al., 2018), characterized by task-driven, problem-oriented, and learning by doing (Schaap et al., 2012; Hämäläinen et al., 2015; Jossberger et al., 2020). Instructional design follows the workflow of problem or task resolution, including the following stages: obtaining information, defining tasks, making plans, making decisions, implementing plans, checking, evaluating, and providing feedback. During this process, students have to navigate the problem-solving logic by defining the task’s content and objectives, making plans to complete the task, selecting the best plan from various options, implementing the plan, checking task completion, and evaluating the effectiveness of the outcome. The specific strategies for conducting blended learning in VET obtained in the above stages are shown in Table 7.

### 4.3. Questionnaire Results on the Effectiveness of Blended Learning Strategies

**Figure 4:**
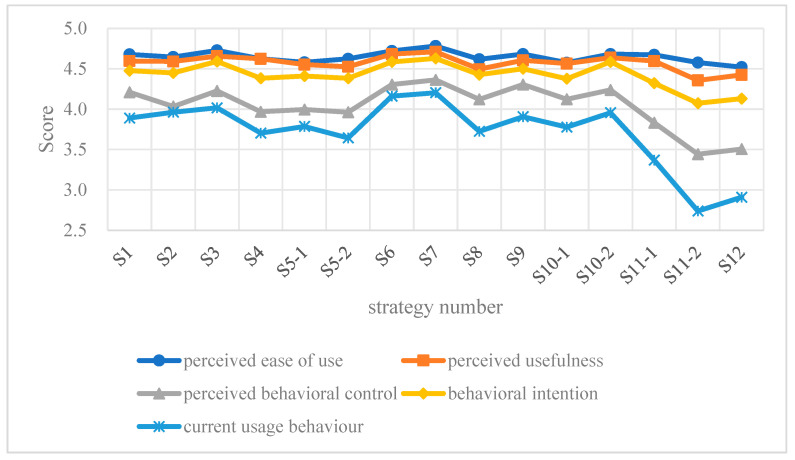
VET teachers’ view of the proposed general blended learning strategies.

Figure 4 illustrates VET teachers’ evaluations of the proposed general blended learning strategies. The dimensions of perceived usefulness (M > 4.3) and perceived ease of use (M > 4.3) demonstrated strong acceptance, while behavioral intention scores consistently exceeded 4.0, indicating a positive perception of the strategy framework. Moreover, the feedback score for current usage behavior was the lowest across all strategies. However, current implementation levels emerged as the least developed aspect, with 12 out of 15 strategic items scoring a range from 2.5 to 4.0. Notably, three strategies exhibited significantly lower adoption rates compared to others: strategy 11-1 and 11-2 (integration of real workplace situations or business mentors into the teaching and learning process) and strategy 12 (flexible grouping and hierarchical teaching). Strategies 11-2 and 12 received average scores below 3, reflecting minimal integration into daily teaching practices.

Table 8 presents VET teachers’ evaluations of specific blended learning strategies, revealing uniformly high scores across perceived usefulness (M > 4.2), ease of use (M > 4.2), and adoption intention (M > 4.2). However, Type3 displayed implementation challenges, with perceived behavioral control (M = 3.92) and current usage (M = 3.62) scoring markedly lower than other categories. It indicates that although teachers recognize the importance of students’ autonomy, they remain uncertain about the extent of autonomy they should grant their students. Consequently, they frequently supplement autonomous tasks with intervention, a practice diverging from award-winning teachers’ approaches that prioritize sustained student-directed learning.

## 5. Discussion

The present study provided a diverse and high-quality reference for understanding blended learning and improving teachers’ practice by analyzing award-winning videos and daily videos. Unlike previous studies, which only focused on general teaching strategies (Jayalath & Esichaikul, 2020; Luo & Zhou, 2024), this study developed a series of general and specific strategies applicable to different types of courses. The perceived usefulness, ease of use, and intention to use these strategies were also assessed among VET teachers through a questionnaire, indicating their practical value in guiding blended learning in higher vocational courses. The results of the general instructional strategies were similar to the strategies proposed in previous studies. For example, previous studies found that strategies such as offering flexible learning opportunities (Vanslambrouck et al., 2018), providing situational support through online resources (Butz & Stupnisky, 2017), using encouraging language (Chiu, 2021), and utilizing process data for timely feedback (Ameloot et al., 2024) remained effective in blended learning contexts for senior high school students, bachelor students, and graduate students. The current study extends the application of these instructional strategies to the blended learning situation for VET students.

The present study summarized instructional strategies applicable to blended learning from a bottom-up perspective, finding that they align well with self-determination theory. Self-determination theory (SDT) posits that students have three basic psychological needs: autonomy, competence, and relatedness (Deci & Ryan, 1987). Recently, many studies have found that teachers’ instructional behaviors in blended learning environments significantly impact students’ learning motivation by influencing their basic psychological needs (Adam et al., 2023; Chiu, 2022; Vanslambrouck et al., 2018).

This study also indicated that teachers should first focus on satisfying students’ needs for autonomy, competence, and relatedness to implement blended learning effectively. For example, when teachers use non-controlling language with open-ended questions and allow students to express their views, students’ psychological need for autonomy will be addressed (Y. Su & Reeve, 2011; Shelton-Strong, 2022). This theoretical framework supports the observed differences in questioning styles between the two types of courses. The following pedagogical recommendations are formed based on the above findings. First, focus on enhancing teacher–student interaction and allow students to communicate freely. It means that teachers should design questions to encourage interaction and enable students to express their thoughts rather than monologic delivery (Y. Su & Reeve, 2011). Second, when engaging with students through questions, teachers should consciously utilize open-ended questions to stimulate deeper thinking. Third, balance questions between group and individual according to their purpose: closed-ended questions may be more suitable for assessing collective understanding, while open-ended questions are more effective for fostering individual in-depth recognition (Lee et al., 2012). While previous studies on teacher–student interaction have highlighted the importance of questioning techniques and communication styles in supporting students’ autonomy (Dukuzumuremyi & Siklander, 2018; Karasova, 2023; Müller et al., 2023), this study demonstrated that these strategies were also effective for VET students.

Moreover, award-winning teachers were found to be more likely to design tasks that are closely aligned with real-world work scenarios than teachers in daily courses. This finding aligns with the recommendation advocated by competence support that teachers boost students’ confidence by providing clear and explicit guidance (Deci & Ryan, 1987; Cui et al., 2022). Based on LSA results about feedback, we also found award-winning teachers more often use encouraging language and emotional incentives when providing feedback, which aligns with the recommendation advocated by relatedness support (Stroet et al., 2013).

Furthermore, considering that fixed steps for blended learning may be too broad to play a practical guiding role in blended learning across different courses, this study found that specific actions within the same stage can vary significantly between courses. For instance, during the planning, decision-making, and implementation stages (see Table 7), the teacher–student dialogue classroom represented by Type1 emphasizes cognitive goals and is teacher-led mainly dialogue. Therefore, teachers should focus on encouraging students to participate in discussions. Type2 and Type3 concentrate on skill goals, but Type2 involves teachers demonstrating before students practice, while Type3 allows students to explore independently before receiving teacher guidance. At these stages, the former prioritizes how teachers facilitate learning through demonstration, whereas the latter emphasizes providing flexible guidance and feedback. The above findings reveal a consistent pattern: VET teachers exhibit positive perceived usefulness, perceived ease of use, and behavioral intention for both general and specific strategies, but their current usage behavior is not ideal. These results validate the necessity of the proposed instructional strategies for bridging the theory–practice gap in blended learning implementation. These findings also hold practical implications for future professional development programs targeting VET teachers’ implementation of blended learning. Specifically, teachers can select appropriate blended learning models based on their course types and instructional needs and receive more tailored training in instructional strategies.

## 6. Conclusions, Implications, and Limitations

The present evidence-based study systematically derived blended learning strategies for Chinese vocational education by comparing 53 award-winning courses and 40 daily course videos, complemented by teachers’ feedback on the dimensions of usefulness, ease of use, and willingness to use these strategies through questionnaires. A series of strategies was developed to guide teachers in effectively implementing blended learning (shown in Table 6 and Table 7), answering the research questions posed in this study. We found that the general strategies were closer to the theoretical guidance of SDT, while the specific strategies were guided by action-oriented learning. It is important to note that the strategies proposed in this study are not intended to be used in all courses but rather assembled strategies for teachers to adopt flexibly according to their needs.

The main implications of the present study are as follows. Firstly, while blended learning efficacy is well-established in basic education (Dey & Bandyopadhyay, 2019; Ye et al., 2022) and higher education (Castro, 2019; van der Stap et al., 2024), this study addresses a critical gap in vocational education research, particularly within China’s unique vocational student population. Secondly, while existing research has proposed some broad instructional strategies for blended learning (Müller et al., 2023; van der Stap et al., 2024), this study broadens the scope by examining different types of VET courses. We have proposed general and specific strategies applicable to different courses, which can assist teachers in implementing blended learning more effectively. Thirdly, the study reveals that teaching strategies identified based on award-winning experiences can be categorized and explained using the SDT. This finding encourages researchers to explore the application of SDT in blended learning within vocational education, emphasizing the importance of providing students with autonomy support, competence support, and emotional support. Therefore, the strategies developed in this study are more systematic and comprehensive.

This study also has some limitations that should be noted. The number of videos in the present study is significantly larger than those examined in previous studies (D. Wu et al., 2022). The award-winning videos included in the analysis cover all major categories and provincial administrative units to ensure a representative sample. However, since the teaching and learning process is inherently complex and varies widely, future studies should incorporate more blended learning videos from different regions, subject areas, and varying teacher abilities to better analyze results and test the generalizability and applicability of the findings. It should be noted that this study relied on questionnaire data to evaluate teachers’ perceptions of the proposed instructional strategies. Although an open-ended question was included to collect detailed feedback and suggestions, the responses received were generally seldom and generic, such as “good” or “very useful”. As a result, only the results from the closed-ended items were reported in the findings section, and there remained a lack of in-depth qualitative data regarding teachers’ actual perceptions and implementation intentions. Future research could address this limitation by conducting interviews with award-winning and frontline VET teachers to gather richer insights, which would help further refine and enhance the proposed strategies.

## Figures and Tables

**Table 1:** The duration of the videos for various courses.

Types of Courses	Type1	Type2	Type3
Award-winning courses	138,967 s	69,327 s	17,172 s
Daily courses	106,476 s	27,690 s	25,993 s

**Table 2:** Video analysis coding framework for blended learning in VET course.

Dimension	Category		Coding Indicators
Teacher– student interaction	Teacher-to-student interaction		Teacher operation (TS1) Teacher relates content to work context (TS2) Teacher expresses ideas about content (TS3) Teacher states content (TS4) Teacher states rules and action requirements (TS5)
	Student-to-teacher interaction		Student presentation (ST1)
	Teacher– student bidirectional interaction	Individual interaction (1 to 1)	Student operation while teacher gives personalized guidance (TSI1) Student discussion while teacher gives personalized guidance (TSI2)
		Social interaction (1 to N)	Authoritative discussion (teacher asks and answers themselves and students respond with yes or no) (TSG1) Teacher-led dialogue (teacher asks and students respond) (TSG2) Student-led dialogue (students ask and teacher responds) (TSG3) Student presentations interspersed with teacher comments (TSG4) Teacher feedback on students’ previous responses (TSG5) Teacher gives emotional expression (e.g., praise) for student performance (TSG6) Teacher gives classroom prompts while students operate (no direct guidance) (TSG7) Students operate under the guidance of the teacher (TSG8)
Student–student interaction			Student discussion (SS1) Role-playing (SS2) Peer assessment (SS3)
Student and content interaction			Student operation (teacher non-participant behavior) (SC1) Students complete homework (teacher non-participant behavior) (SC2) Students view information (teacher non-participant behavior) (SC3)
Others			Teacher discourse unrelated to content (e.g., maintaining order) (O1) Silence that does not contribute to the lesson (O2) Silence that contributes to the lesson (e.g., thinking) (O3) Student signing in (O4)

**Table 3:** Details of the participants in the questionnaire.

		Sample Size (N)	Percentage
Gender	Male	57	31.1%
	Female	126	68.9%
Age	Lower than 26 years old	2	1.1%
	26–40 years old	117	63.9%
	41–55 years old	57	31.1%
	More than 55 years old	7	3.8%
Academic title	Assistant lecturer	5	2.7%
	Lecturer	95	51.9%
	Associate professor	41	22.4%
	Professor	30	16.4%
Years of teaching experience	Less than 1 years	5	2.7%
	1–3 years	32	17.5%
	4–6 years	21	11.5%
	7–10 years	25	13.7%
	11–20 years	82	44.8%
	More than 20 years	18	9.8%

**Table 4:** Results of three question-and-answer activities in various instructional videos.

		Closed-Ended Questions from Teachers		Open-Ended Questions from Teachers		Questions from Students		F	*p*
		Time Period (s)	Percentage	Time Period (s)	Percentage	Time Period (s)	Percentage		
Type1	AC	305	0.113	554	0.205	105	0.039	53.04	<0.001
	DC	373	0.138	356	0.132	46	0.017		
Type2	AC	81	0.030	176	0.065	70	0.026	42.23	<0.001
	DC	68	0.025	32	0.012	19	0.007		
Type3	AC	27	0.010	86	0.032	76	0.028	94.56	<0.001
	DC	57	0.021	127	0.047	0	0		

Note: AC is award-winning courses; DC is daily courses. Type1: teacher–student dialogue; Type2: teacher demonstration; Type3: student-independent operation.

**Table 5:** Z-score results for the behavioral sequence.

Behavioral Sequence	Type1		Type2		Type3	
	AC	DC	AC	DC	AC	DC
TS2 → TS3	3.01 *	−0.2	2.51 *	−0.2	2.74 *	−0.4
TSG5 → TSG6	−0.94	0.35	0.2	−0.25	3.43 *	−0.56
TSG6 → TSG5	7.17 *	0.42	4.83 *	−0.25	1.73	−0.56

Note: TS2: Teacher relates content to work context; TS3: Teacher expresses ideas about content; TSG5: Teacher feedback on students’ previous responses; TSG6: Teacher gives emotional expression (e.g., praise) for student performance. * indicates statistical significance at the 95% confidence level (*p* < 0.05) for the Z-score values.

**Table 6:** Universal blended learning strategies.

Stage		Teaching Strategy	Situation	No.	Explanation of Teacher Actions	SDT
During class	Introduce	Focus on the link between classroom activities and pre-course activities.	T	1	At the beginning of each class, teachers should offer explanations and feedback on student activities and their results, bridging the connection between in-class and out-of-class and online and offline learning experiences.	CS
		Help students understand the connection between what they have learned and the workplace and enhance the attractiveness of the tasks by relating them to local features or daily life.		2	Teachers should connect lessons to real-world applications in the workplace and daily life, helping students realize the situations and problems the lessons can assist them with and motivating their active participation.	AS RS
	Teaching new knowledge	Focus on teacher–student interaction, encouraging student participation by emphasizing the importance of the activity and providing opportunities to express themselves and communicate.	F	3	Teachers need to encourage teacher–student and student–student interaction rather than one-way information delivery. E.g., clearly explaining the purpose and meaning of the activity to enhance student engagement, emphasizing opportunities for self-expression, and questioning during the process.	
	Issuing tasks	Design tasks and problems orientated to the needs of real jobs, including clear explanations of the rules and requirements.	T	4	The tasks or problems presented should be closely related to practical application, and the rules should be clearly stated to regulate students’ actions. It aims to motivate them to integrate what they have learned with the needs of real workplaces and prepare them for future employment.	CS
	Implementation	Offer students well-structured guidance.		5-1	Teachers should provide relevant support and scaffolding when students operate.	
				5-2	Offering timely, targeted, and personalized guidance is crucial, rather than delayed and assumptive group feedback.	
	Feedback	Feedback reflects encouragement of emotions.	F	6	When giving feedback on students’ answers, teachers should pay attention to providing more emotional incentives and more encouraging language to students.	CS RS
		Feedback reflects an attitude of equal respect for students.		7	Instead of offering vague praise like “good” or “very good,” teachers should combine the content and students’ performance to provide clear and constructive feedback to prevent negative feelings associated with superficial praise.	
		Design the feedback process flexibly to support assessment for learning and the integration of knowledge and evaluation.	T	8	After the student-led activities, teachers should first guide students to evaluate each other’s performances and then give feedback on students’ responses and mutual assessment to strengthen cognition and meta-cognition.	CS AS RS
	Extension	Allow sufficient time for students to consolidate their understanding and make improvements.	F	9	Allow time for problem-solving and consolidation following the feedback on students’ actions.	CS AS
Throughout all phases of classroom		Employ a variety of questioning styles during teacher–student interactions.		10-1	Balance questions between group and individual contexts based on the questioning purpose: memorizing and closed questions are suitable for assessing collective understanding, while speculative and open-ended questions are suitable for individual in-depth understanding.	AS
				10-2	Teachers should consciously use open-ended questions to stimulate students’ thinking during interaction.	
		Integrating real workplace situations or business mentors into the school’s teaching and learning process.	T	11-1	Show videos featuring presentations about companies or artisans to motivate students and illustrate job requirements and professionalism.	CS RS
				11-2	Engage with graduates who have entered the workforce, connecting with them remotely to participate in feedback coaching on student presentations or contact them after class to record feedback videos.	
		Use flexible grouping strategies and hierarchical teaching methods.	F	12	Differentiate teaching according to the student’s situation: provide varying guidance levels and assign tasks with differing difficulty levels. Pay attention to the diverse levels within student groups, name them with motivational terms, and timely adjust the grouping accordingly to incentivize students who have progressed to a higher level.	

Note: AS is autonomy support; CS is competence support; RS is relatedness support; T is technology-supported classroom environments; F is face-to-face environment.

**Table 7:** Specific blended learning strategies.

**Stage**	**Type1**	**Type2**	**Type3**
Obtaining information	⯎ Assigning tasks for students to research information in advance enhances their sense of participation.	⯎ Utilizing information technology flexibly to help connect learning to real-world scenarios. ⯎ Employing the use of work-related cases to enhance students’ professional quality by showcasing role models.	
Defining tasks	⯎ The teacher explains the task’s requirements, including the purpose, rules, and considerations.		⯎ The task is divided into sub-tasks that students can explore independently, with the situation’s complexity increasing over time. These tasks are often related to student’s daily lives and gradually reinforced.
Making plans	⯎ In the process of information transfer, the primary engagement shifts from the teacher to the students and then back to the teacher, fostering interaction between them. ⯎ The teacher’s role evolves through three stages: as an instructor in the early phase, a guide in the middle phase, and an inducer in the latter phase.	⯎ During the operation demonstration, teachers incorporate numerous dialogues with students to enhance their understanding of the task rather than a one-way communication model where the teacher lectures and the students passively receive information. ⯎ When demonstrating operations, teachers often utilize large-screen projections, live broadcasts, and other tools to ensure students can see the process clearly. Additionally, they may record operation videos in advance so students can watch the demonstrations conveniently. ⯎ When students operate, teachers change the task situation so that students do not simply imitate the operation steps demonstrated by teachers but strengthen their cognition and understanding through hands-on experience.	⯎ Throughout the process, the teacher provides feedback on issues encountered: (1) If it is a common problem, the teacher should interrupt in time, allowing all students to continue working after uniform feedback. (2) If it is an individual but typical problem, students can be given enough space for trial and error and reflection before uniform feedback.
Making decisions			
Implementing plans			
Checking	⯎ In cases where students are not actively participating and the classroom falls silent, teachers should offer timely guidance to reduce the difficulty of the questions, thereby avoiding scenarios where teachers engage in self-questioning, self-answering, and one-way communication.		⯎ Teachers should prepare the lesson in advance, organize the process of inquiry and presentation in a timely and effective, and avoid situations where students wait due to ineffective classroom management.
Evaluation and feedback	⯎ Often conducted in the form of student presentations: (1) After the presentation, students evaluate their peers, and then the teacher gives feedback on both the presentation and peer evaluation. (2) The teacher should provide a timed evaluation after each group’s presentation rather than a collective evaluation after all groups have presented.	⯎ After practicing, students engage in peer evaluations and ask each other questions, followed by the teacher’s feedback. ⯎ Teachers’ feedback is not the end, and they often assign extension tasks to ensure that students understand and can apply the feedback provided.	
Consolidate and improvement	⯎ Link to the work situation, thus enhancing students’ cognitive and professional understanding between learning content and actual work needs.	⯎ To facilitate growth, it is important to gradually increase the difficulty of tasks while reducing the available scaffolding.	

**Table 8:** VET teachers’ view of the proposed specific blended learning strategies.

	Type1		Type2		Type3	
	M	SD	M	SD	M	SD
perceived ease of use	4.75	0.49	4.67	0.56	4.74	0.47
perceived usefulness	4.68	0.51	4.54	0.69	4.64	0.58
perceived behavioral control	4.19	0.87	3.91	1.15	4.24	0.83
behavioral intention	4.51	0.68	4.25	0.99	4.49	0.65
current usage behavior	4.01	0.98	3.62	1.21	3.99	1.06

Note: M = mean; SD = standard deviation.

## Data Availability

The data in this study can be provided upon request by sending an e-mail to the corresponding author.

## Appendix A

**Table A1:** Behavioral sequences for three course categories (award-winning vs. daily instruction).

	Award-Winning Courses	Daily Courses
Type1 teacher– student dialogue		
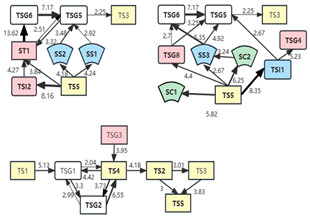
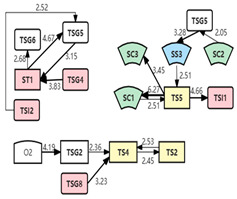
Type2 teacher demonstration		
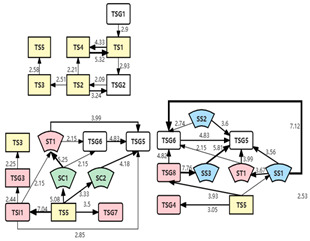
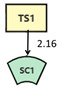
Type3 student- independent operation		
